# *TractoInferno* - A large-scale, open-source, multi-site database for machine learning dMRI tractography

**DOI:** 10.1038/s41597-022-01833-1

**Published:** 2022-11-25

**Authors:** Philippe Poulin, Guillaume Theaud, Francois Rheault, Etienne St-Onge, Arnaud Bore, Emmanuelle Renauld, Louis de Beaumont, Samuel Guay, Pierre-Marc Jodoin, Maxime Descoteaux

**Affiliations:** 1 University of Sherbrooke, Computer Science Department, Sherbrooke, J1K 2R1 Canada; 2 Montreal Sacred-Heart Hospital Research Centre, Montreal, H4J 1C5 Canada; 3 University of Montreal, Department of Surgery, Montreal, H3C 3J7 Canada; 4 Imeka, Sherbrooke, J1H 4A7 Canada

**Keywords:** Computational neuroscience, Computer science, Neural circuits

## Abstract

*TractoInferno* is the world’s largest open-source multi-site tractography database, including both research- and clinical-like human acquisitions, aimed specifically at machine learning tractography approaches and related ML algorithms. It provides 284 samples acquired from 3 T scanners across 6 different sites. Available data includes T1-weighted images, single-shell diffusion MRI (dMRI) acquisitions, spherical harmonics fitted to the dMRI signal, fiber ODFs, and reference streamlines for 30 delineated bundles generated using 4 tractography algorithms, as well as masks needed to run tractography algorithms. Manual quality control was additionally performed at multiple steps of the pipeline. We showcase *TractoInferno* by benchmarking the learn2track algorithm and 5 variations of the same recurrent neural network architecture. Creating the *TractoInferno* database required approximately 20,000 CPU-hours of processing power, 200 man-hours of manual QC, 3,000 GPU-hours of training baseline models, and 4 Tb of storage, to produce a final database of 350 Gb. By providing a standardized training dataset and evaluation protocol, *TractoInferno* is an excellent tool to address common issues in machine learning tractography.

## Abstract (summary)

**Table:** 

Measurement(s)	Diffusion Weighted Imaging • Magnetic Resonance Imaging of the Brain without Contrast • Diffusion Tensor Imaging
Technology Type(s)	3 T MRI scanner
Factor Type(s)	Age • Gender
Sample Characteristic - Organism	Homo sapiens

## Background & Summary

Tractography is the computerized process of reconstructing brain white matter fibers from diffusion MRI (dMRI) data. It usually consists of four steps: (i) pre-processing diffusion-weighted images (DWI), (ii) estimating local fiber directions, (iii) reconstructing white matter pathways (i.e. tractography), and (iv) delineating fiber bundles^1,2^.

Current “traditional” tractography approaches (deterministic and probabilistic) mostly rely on making local point-wise decisions in the fiber Orientation Distribution Function (fODF) field, iterating until termination^3,4^. Global methods have also been proposed^5–8^, but (Rheault *et al*.)^9^ mentions that “[…] global tractography methods ultimately rely on local information patched together” and “even global tractography algorithms struggle to correctly assemble a streamline”. Tractogram filtering^10–13^ is a popular post-processing method used to remove streamlines that do not fit anatomical constraints (such as explaining the underlying signal), but requires an over-complete tractogram as it does not create new streamlines, thus effectively “wasting” computing power. Finally, streamline clustering^14,15^ can be used to group streamlines based on similarity and remove outliers, but it suffers from the same drawback as tractogram filtering, as it requires an over-complete tractogram.

These approaches mostly rely on mathematical models or anatomical priors, and do not require histological ground truth to work. However, this is an issue for machine learning algorithms, where the training dataset is an integral part of the resulting model^16^. Machine learning methods need reference streamlines to train on. Unfortunately, on real datasets, streamlines can only be generated by traditional tractography methods, which are imperfect by their very nature^2^. This is an issue for testing if the predictions made by these methods are reliable or not. Luckily, by combining streamlines (both true positives and false positives) generated by several tractography algorithms and using filtering and clustering to remove as much false positives as possible, it is possible to establish a *gold standard* reference dataset. Even without a histologically accurate ground truth, it would be desirable to have algorithms that can reproduce a gold standard reference while generating as little false positive streamlines as possible.

In the recent years, machine learning (ML) algorithms have been proposed to improve the tractography process by some combination of (i) using the full diffusion information, (ii) generating more reliable streamlines using a reference teacher dataset, or (iii) integrating spatial context to guide the tracking process (either neighbourhood or path information)^16–20^. Unfortunately, these machine learning methods train and evaluate their models on different dataset which makes it difficult to compare their true generalization capabilities^16^. Additionally, data pre-processing vary between proposed methods, and different algorithms and protocols are used to generate the reference tracts. Finally, evaluating the true generalizability of a model is almost impossible without diverse (aka multi-site) training and test sets. As a result, those discrepancies in methodology make it very challenging to assess the reliability of a single approach, and make it impossible to fairly compare algorithms against one another.

To our knowledge, there are few datasets that contain both diffusion MRI and gold standard tractography, and none that include multiple sites. Proposed methods in the existing literature usually use in-house (private, ad-hoc) tractography datasets to train their models, often subjects from the HCP database. (Poulin *et al*.)^16^ provides a more detailed review of existing tractography datasets and their limitations.

We propose to address this problem by building *TractoInferno*: the largest publicly available, multi-site, dMRI and tractography database, which provides a new baseline for training and evaluating machine learning tractography methods. It provides 284 samples acquired from 3 T scanners across 6 different sites. *TractoInferno* includes T1-weighted images, single-shell diffusion MRI (dMRI) acquisitions, spherical harmonics fitted to the dMRI signal, fODFs, and reference streamlines for 30 delineated bundles generated by combining 4 different tractography algorithms, as well as masks needed to run tractography algorithms.

We use *TractoInferno* to benchmark the 4 tractography algorithms used to create the reference tractograms, along with the learn2track^18^ algorithm and 5 variations of the same recurrent neural network architecture, inspired in part by the models of (Benou & Riklin-Raviv)^21^ and (Wegmayr *et al*.)^20^. Creating the *TractoInferno* database required approximately 20,000 CPU-hours of processing power, 200 man-hours of manual QC, 3,000 GPU-hours of training baseline models, and 4 Tb of storage, to produce a final database of 350 Gb.

*TractoInferno* is a dataset intended to promote the development of ML tractography algorithms, which generally suffer from multiple issues, such as limited datasets or inconsistent training data. Its large-scale and multi-site aspect is an undeniable benefit to best evaluate the generalization capabilities of new ML algorithms. We consider *TractoInferno* to be by far the best available tool for training, evaluating, and comparing future ML tractography algorithms.

## Methods

### Datasets

The proposed dataset is made of a combination of six dMRI databases, either publicly available or acquired through open-access data sharing agreements, and free to redistribute under a Creative Commons CC0 license. Databases were chosen with the explicit goal of having a diversity of scanner manufacturers, models, and protocols. We chose to fix certain parameters for uniformity, such as having only healthy subjects, acquired on 3 T scanners, and using b-values of around 1000 s/mm^2^, as we don’t know how they could affect machine learning models. The focus is effectively on assessing the reliability of algorithms under different scanner manufacturers and acquisition protocols. We obtained an initial number of data from 354 subjects, with the original metadata described in Table 1.

**Table 1:** Original datasets metadata. Not all metadata information was available from the original datasets.

Name	*Mazoyer et al*.^22^	*Tsushida et al*.^23^	*DeLuca et al*.^25^	*Poldrack et al*.^27^	*Tamm et al*.^28^	*Tremblay et al*.^30^
**Scanner**	3 T Philips Achieva	3 T Siemens Prisma	3 T Siemens Prisma	3 T Siemens Trio	3 T GE Discovery MR750	3 T Siemens Magnetom TIM Trio
**# subjects**	39	20	64	130	86	15
**Age avg**	28.1	21.4	31.9	31.3	N/A	58.1
**Age std**	7.3	1.7	7.6	8.7	N/A	5.3
**F/M**	0/39	10/10	49/15	62/68	44/42	0/15
**L/R**	8/31	N/A	0/64	N/A	N/A	3/12
**Resolution**	2	1.75	2	2	2.3	2
**b-value**	1000	1000	1000	1000	1000	700
**TR**	8500	3540	1800	9000	7000	9200
**TE**	81	75	70	93	81	84
**Nb dirs**	21*	32	128**	64	45	30

Missing metadata is reported as {N/A}. Resolution is in mm^3^ isotropic. b-value is in s/mm^2^. TR and TE are in ms.

*21 directions acquired twice by reversing the gradient polarity, then averaged over another identical acquisition (total of 84 DWI volumes).

**64 directions acquired twice, not averaged.

#### Mazoyer et al. - BIL & GIN

We retained 39 subjects from the BIL&GIN database^22^, acquired on a 3 T Philips Achieva, with the following dMRI protocol: TR = 8500 ms, TE = 81 ms, angle = 90°, SENSE reduction factor = 2.5, FOV 224 mm, acquisition matrix 112 × 112, 2 mm^3^ isotropic voxel.

The dMRI acquisition consisted of 21 gradient directions at b = 1000 s/mm^2^, acquired twice by reversing the polarity, and then repeated twice for a total of 84 DWI images, averaged down to a single volume with 21 directions. A single b = 0 s/mm^2^ was also acquired alongside the DWI images. Subjects were all males, with age mean/std of 28.1 + -7.3 (Min: 20, Max: 57). 8 subjects were left-handed and 31 right-handed.

All participants gave written consent prior to participation in the study, which was approved by the local ethics committee (CCPRB Basse-Normandie).

#### Tsushida et al. - MRi-Share

We obtained 20 subjects from the MRi-Share database^23^, acquired on a 3 T Siemens Prisma, with a dMRI protocol designed to emulate the UKBioBank project^24^, specifically: TR = 3540 ms, TE = 75 ms, 1.75 mm^3^ isotropic voxel.

We selected the b = 1000 s/mm^2^ DWI images only, consisting of 32 gradient directions, and 3 provided b = 0 s/mm^2^ images. Subjects were composed of 10 females, 10 males, with age mean/std of 21.4 + -1.7. Minimum/maximum age and handed-ness metadata were not available.

The MRi-Share study protocol was approved by the ethics committee (CPP2015-A00850-49), and all participants signed an informed written consent form.

#### DeLuca et al. - Bilingualism and the brain

We have 64 subjects from the *Bilingualism and the Brain* database^25,26^, acquired on a 3 T Siemens Prisma, with the following dMRI protocol: Echo planar imaging, TR = 1800 ms, TE = 70 ms, acquisition matrix 256 × 256, 2 mm^3^ isotropic voxel.

The dMRI acquisition consisted of 64 gradient directions at b = 1000 s/mm^2^, acquired twice, and 4 b = 0 s/mm^2^ images. Subjects were composed of 49 females and 15 males, with age mean/std of 31.9 + -7.6 (Min: 18, Max: 52). All subjects were right-handed.

The research procedures in this study were approved by the University of Reading Research Ethics Committee. Before taking part in the experiment, participants gave written informed consent and confirmed no contraindication to MRI scanning.

#### Poldrack et al. - UCLA CNP

We got 130 healthy subjects from the *UCLA Consortium for Neuropsychiatric Phenomics LA5c Study*^27^, acquired on a 3 T Siemens Trio, with the following dMRI protocol: echo planar imaging, TR = 9000 ms, TE = 93 ms, acquisition matrix 93 × 93, 90 degree flip angle, 2 mm^3^ isotropic voxel. DWI were corrected for eddy currents and head motion using the b0 images as reference.

The dMRI acquisition consisted of 64 gradient directions at b = 1000 s/mm^2^, and 1 b = 0 s/mm^2^ image. Subjects consisted of 62 females and 68 males, with age mean/std of 31.3 + -8.7 (Min: 21, Max: 50). Handed-ness metadata was not available.

Participants of this study gave written informed consent following procedures approved by the Institutional Review Boards at UCLA and the Los Angeles County Department of Mental Health.

#### Tamm et al. - The stockholm sleepy brain study

We retained 86 subjects from the Stockholm Sleepy Brain Study database^28,29^, acquired on a 3 T GE Discovery MR750, with the following dMRI protocol: Echo planar imaging, TR = 7000 ms, TE = 81 ms, 2.3 mm^3^ isotropic voxel.

The dMRI acquisition consisted of 45 gradient directions at b = 1000 s/mm^2^, along with 5 b = 0 s/mm^2^ images. Subjects were composed of 44 females and 42 males, with 47 subjects in the [20–30] years old bracket and 39 subjects in the [65–75] years old bracket. Handed-ness was not available.

This study was approved by the Regional Ethics Review board of Stockholm (2012/1870-32), and all participants gave written informed consent.

#### Tremblay et al. - mTBI and Aging study (controls)

We obtained 15 subjects from the mTBI and Aging Study^30^, all controls from the “remote” group. They were acquired on a 3 T Siemens Magnetom TIM Trio, with the following dMRI protocol: TR = 9200 ms, TE = 84 ms, 2 mm^3^ isotropic voxel.

The dMRI acquisition consisted of 30 gradient directions at b = 700 s/mm^2^. along with 1 b = 0 s/mm^2^ image. Subjects were all males, with age mean/std of 58.1 + -5.3 (Min: 52, Max: 67). 3 subjects were left-handed and 12 were right-handed.

All participants provided written informed consent in accordance with the “Comité d’éthique de la recherche vieillissement-neuroimagerie du CIUSSS du Centre-Sud-de-l’île-de-Montréal” of the CRIUGM (Montréal, H3W 1W5, Canada).

### Data processing

**Fig. 1:**
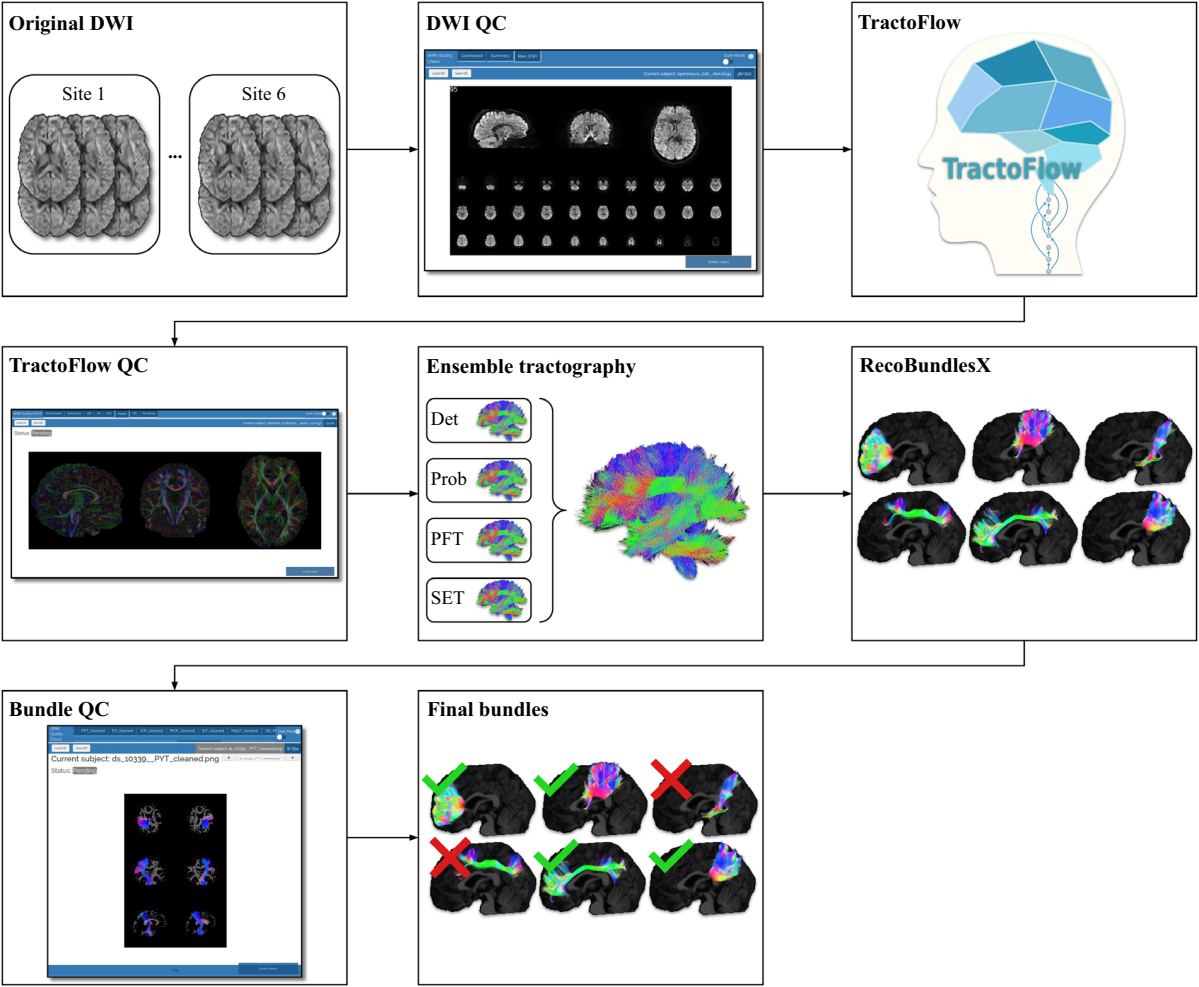
*TractoInferno* processing pipeline, from original DWI images to final bundles.

We processed the original acquisition volumes of the 354 aforementioned subjects with the same pipeline to offer a uniform database of dMRI images, derivatives, and bundle tractograms. First, all original DWI went through a manual quality control (QC) step to remove any obvious errors prior to the processing pipeline. In this case, QC is done by a thorough visual inspection of all modalities, along with a spherical representation of the acquisition scheme. Then, the *TractoFlow* pipeline was run to process the data and compute necessary derivatives^31–33^. Another QC step was executed afterwards, to remove images with artifacts that could not be corrected automatically. Next, ensemble tractography was performed using four different algorithms to extract a diverse set of streamlines: deterministic tractography^34^, probabilistic tractography^35^, Particle-Filtered Tractography^36^ and Surface-Enhanced Tractography^37^. RecoBundlesX (RBX) was used subsequently to perform bundle extraction on the whole-brain tractograms, using the default suggested bundle models^38,39^. A final manual QC step was performed to examine the extracted bundles, and remove anything that contained obvious mistakes, or did not meet our criteria for bundle extraction. All manual quality control steps were done using dmriqcpy (https://github.com/scilus/dmriqc_flow). Figure 1 shows the processing steps of *TractoInferno*.

From the initial 354 volumes, after all the processing steps and quality control, we were left with 284 volumes and associated bundles. The final volumes were split into training, validation and test sets with a 70%/20%/10% split for reproducibility across future experiments. References to software used in the processing pipeline are provided in Table 6. For a final dataset size of 350 Gb, we needed approximately 20,000 CPU-hours of processing time (using a cluster of nodes, each with 40 cores across 2 Intel Gold 6148 Skylake CPUs at 2.4 GHz), 200 man-hours of manual QC, and 4 Tb of storage. The benchmarked recurrent models also required an additional 3,000 GPU-hours (using NVidia V100SXM2 GPUs with 16 Gb VRAM) for training and generating candidate tractograms. In the next sub-sections, we detail the TractoInferno processing steps.

#### Raw data QC

**Fig. 2:**
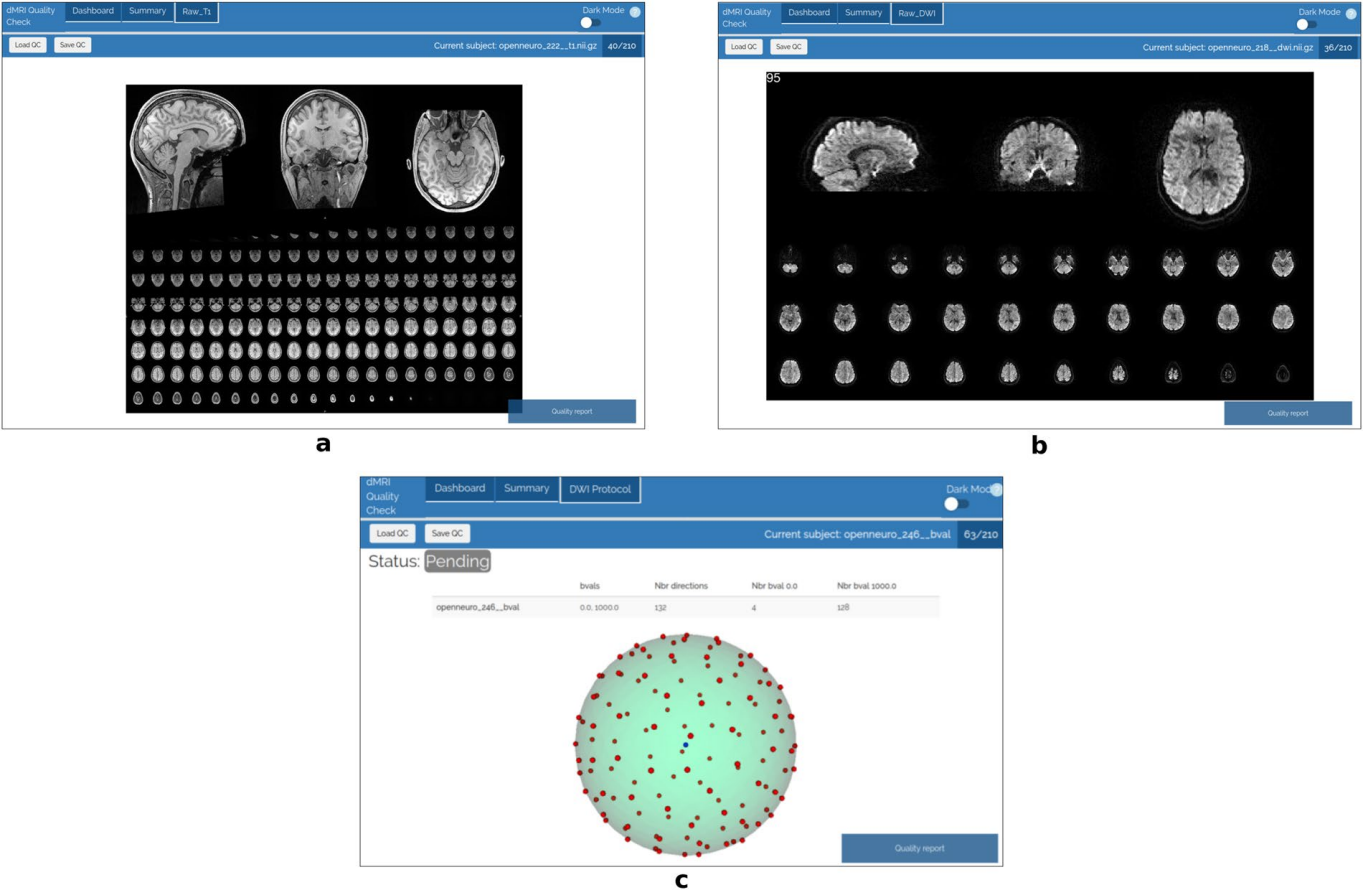
Examples of HTML pages generated by dmriqcpy for data QC. (**a**) 3 slices of the T1 image (one for each axis), plus a mosaic of multiple axial slices; (**b**) 3 GIFs of the dMRI (one slice in each axis), plus a mosaic of multiple axial slices; (**c**) The gradient directions represented on a sphere.

**Fig. 3:**
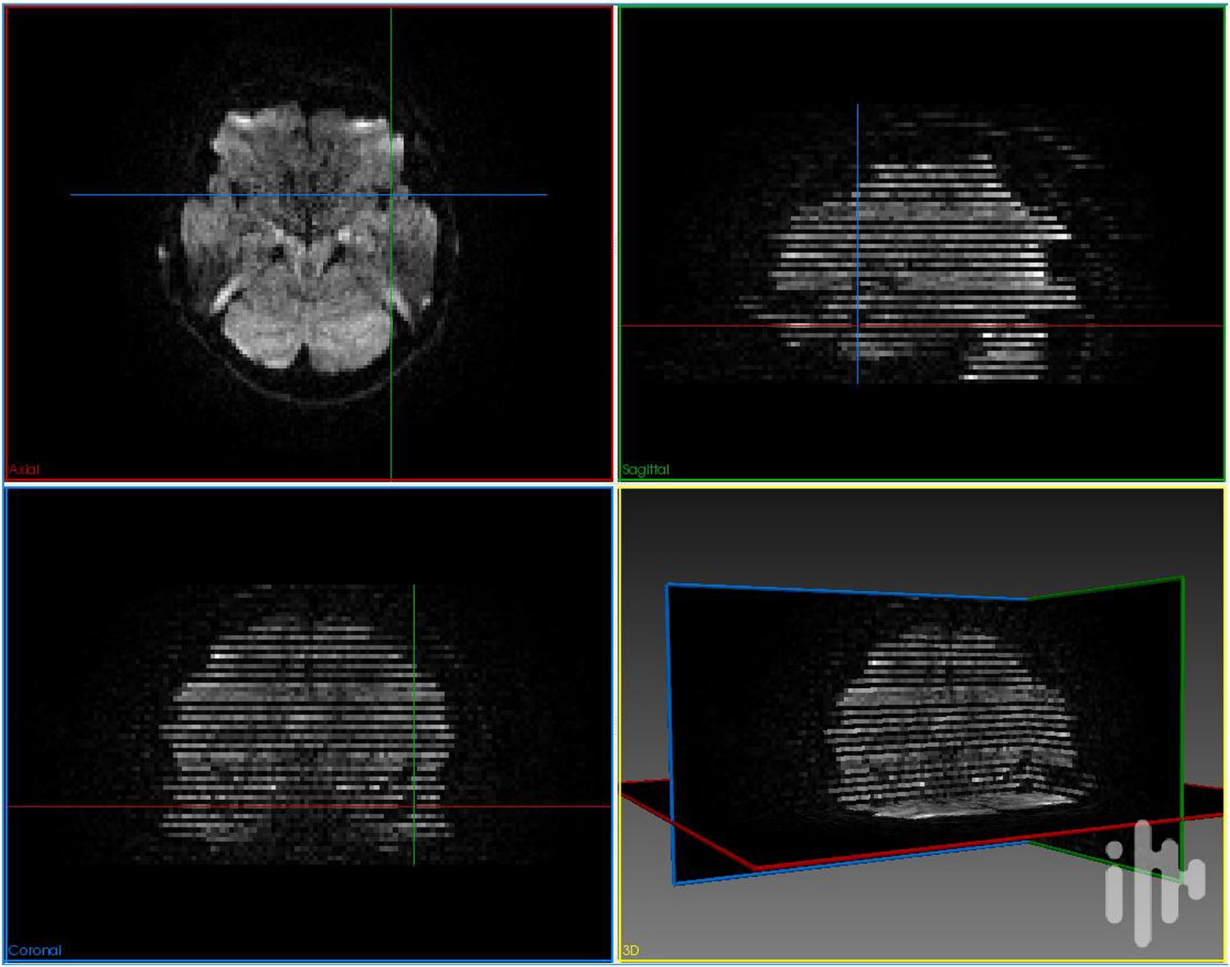
Example of a raw DWI sample that did not pass manual QC because of a slice-drop artifact.

**Fig. 4:**
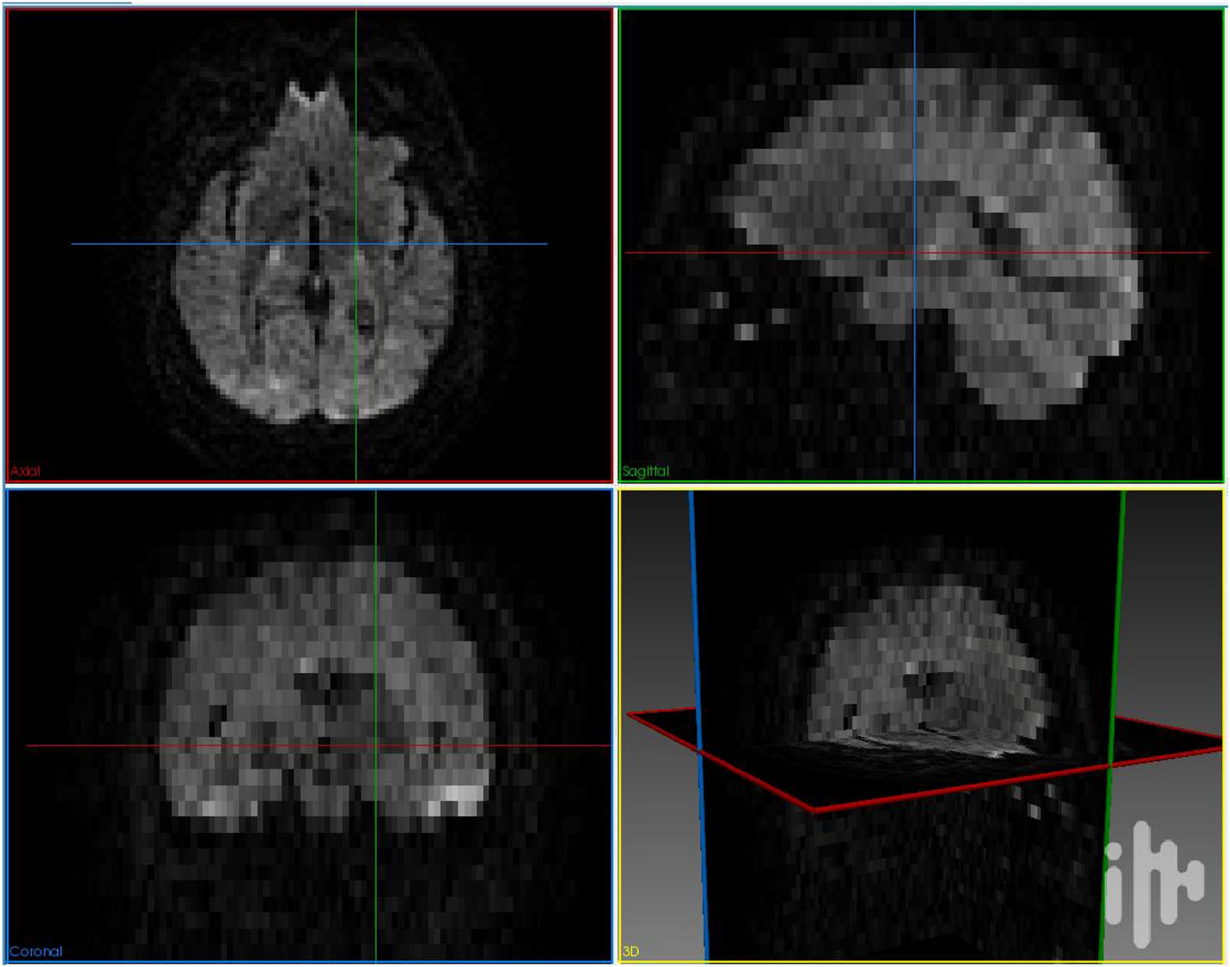
Example of a raw DWI sample that did not pass manual QC because of an acquisition protocol error.

We used *dmriqcpy* to generate QC reports. These reports are in HTML format so it is easily assessed and annotated by multiple people. The raw data reports contain multiple tabs with complementary information, as shown in Fig. 2. Three different raters went through the QC reports and individually rated every acquisition with a “score” (either *pass, fail*, or *warning*) and comment if necessary. Specifically, failure cases included the presence of visual artifacts (e.g. missing slices, low signal-to-noise ratio, corrupted data, high spatial distortion) and other artifacts harder to identify (such as a “broken” gradient acquisition scheme). Representative samples of failure cases are shown in Figs. 3 and 4. Afterwards, all subjects tagged as “fail” were removed, and considered as impossible to repair with our available tools. All subjects tagged as “pass” or “warning” were passed on for TractoFlow, the next step in the pipeline. Subjects tagged as “warning” were re-examined after the TractoFlow processing to examine if any issues remained, or if they were compensated for by the pipeline.

**Fig. 5:**
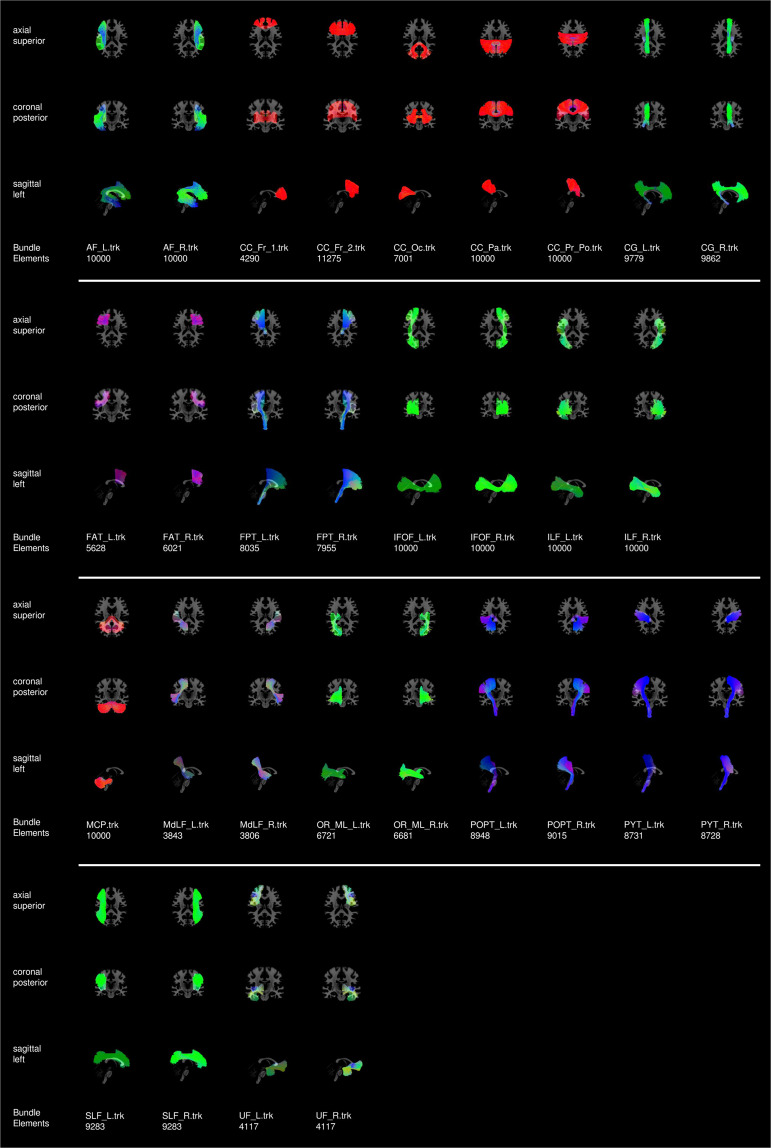
Atlas of bundles used to build *TractoInferno* and evaluate candidate tractograms.

#### TractoFlow pipeline

We used TractoFlow 2.1.1^31^ to process the raw DWI. To make sure that every processing step was traceable and reproducible, a Singularity^32^ image was used along with the Nextflow pipeline^33^. Note however that some results may not be 100% reproducible due to the uncertain nature of registration, parallel processing, and floating point precision. We ran the full pipeline except for the *Topup* process, as not all reverse b0 images were available^40^. Specifically, the pipeline executed the following steps:

- DWI brain extraction^41^, denoising^42^, eddy current correction^43^, N4 bias field correction^44^, cropping, normalization^45,46^, and resampling^47^;
- T1 denoising^48^, N4 bias field correction^44^, registration^49^ and tissue segmentation^50^ maps for Particle-Filtered Tractography^36,51^;
- DTI fitting and metrics extraction^52^;
- fODF fitting using constrained spherical deconvolution^53–55^, with a fiber response function fixed manually to [0.0015,0.0004,0.0004].

#### TractoFlow results QC

Outputs from TractoFlow went through a manual QC pass to identify failure cases. Using *dmriqcpy*, we were able to easily and quickly look at all maps derived from DTI and fODF metrics, along with T1 registration overlay. For example, RGB maps extracted from DTI metrics allowed us to quickly identify if tensor peaks were well-aligned or if a flip was needed, and T1 registration overlays showed whether too much deformation was present.

#### Ensemble tractography

Using a single tractography method as reference for a machine learning algorithm might induce unwanted biases. To avoid this, we chose to use ensemble tractography by combining 4 different algorithms to generate reference streamlines, namely deterministic^34^, probabilistic^35^, particle-filtered^36^, and surface-enhanced^37^ tractography. We fixed the tracking parameters to the standard default values:

- WM + WM/GM interface seeding
- 10 seeds per voxel (Det, Prob, PFT) or 10,000,000 surface seeds (SET)
- Step size 0.2 mm (Det, Prob, SET) or 0.5 mm (PFT)
- WM tracking mask (Det, Prob) or WM/GM/CSF probability maps (PFT, SET)

After tracking, we used streamline compression^56–58^ in order to save space, which means that streamlines have a variable step size that need to be taken into account by ML tractography algorithms. We detail each algorithm in the following three subsections.

##### Deterministic tracking

Deterministic tracking^34^ chooses the fODF peak most aligned with the previous direction as the next streamline step. It seems better suited to connectomics studies^3^, mainly on account of the low number of false positives it produces. While it may be inadequate for spatial exploration and bundle reconstruction, deterministic tracking essentially produces smooth streamlines that follow the easiest path through the fODF field. Smooth streamlines are likely more desirable for ML algorithms rather than chaotic streamlines that often change directions locally.

##### Probabilistic tracking and particle-filtered tractography

Probabilistic tracking^35^ samples a new streamline direction inside a cone of evaluation aligned with the previous direction, with a probability distribution proportional to the shape of the fODF within the cone.

Particle-Filtered Tractography^36^ is an improvement over probabilistic tracking. It takes as input probability maps for streamline continuation/stopping criteria, and allows to “go back” a few steps when a streamline terminates in a region not included in the “termination-allowed” map.

Both algorithms are better suited for spatial exploration, at the cost of producing much more false positives. They are especially effective for bundle reconstruction, in which case there are anatomical priors about both the endpoints that should be connected and the pathway that should be followed by the bundle.

##### Surface-Enhanced Tracking

Finally, Surface-Enhanced Tracking^37^ is a state-of-the-art tractography algorithm that relies on initializing streamlines in an anatomically plausible way at the cortex, then running a PFT tracking algorithm. Indeed, gyri have been shown to be problematic regions for tractography, where low dMRI resolution can lead to a gyral bias in streamline terminations^59^.

To this end, we computed the WM-GM boundary surface from the T1w image using the *CIVET*^60^ tool and the CBRAIN^61^ platform. Then, SET uses a geometric flow method, based on surface orthogonality, to reconstruct the fanning structure of the superficial white matter streamlines. The output of this flow is used to initialize and terminate a PFT tractography algorithm. The result is a tractogram with improved cortex coverage, improved fanning structure in gyri, and reduced gyral bias.

#### Bundle segmentation with RBX

We used RBX^38,39^ to automatically extract WM bundles. The algorithm works by matching streamlines to an atlas of reference bundles. First, a quick registration step brings the atlas into native space using the atlas FA image. Then, a whole-brain tractogram is compared against the bundles atlas using multiple sets of parameters to extract a fixed set of bundles, listed in Table 2. Finally, a majority voting step (label fusion) extracts the final streamlines for each bundle.

**Table 2:** List of bundles in the default RBX atlas.

AC	Anterior commisure
AF	Arcuate fasciculus
CC_Fr_1	Corpus callosum, Frontal lobe (most anterior part)
CC_Fr_2	Corpus callosum, Frontal lobe (most posterior part)
CC_Oc	Corpus callosum, Occipital lobe
CC_Pa	Corpus callosum, Parietal lobe
CC_Pr_Po	Corpus callosum, Pre/Post central gyri
CC_Te	Corpus callosum, Temporal lobe
CG	Cingulum
FAT	Frontal aslant tract
FPT	Frontopontine tract
FX	Fornix
ICP	Inferior cerebellar peduncle
IFOF	Inferior fronto-occipital fasciculus
ILF	Inferior longitudinal fasciculus
MCP	Middle cerebellar peduncle
MdLF	Middle longitudinal fascicle
OR_ML	Optic radiation and Meyer’s loop
PC	Posterior commisure
POPT	Parieto-occipito pontine tract
PYT	Pyramidal tract
SCP	Superior cerebellar peduncle
SLF	Superior longitudinal fasciculus
UF	Uncinate fasciculus

The whole pipeline was run using a Singularity container^32^ and Nextflow^33^ for reproducibility. It is freely available online (https://github.com/scilus/rbx_flow/), along with a suggested bundles atlas (https://zenodo.org/record/4630660#.YJvmwXVKhdU)^62^.

#### Bundle segmentation QC

##### Automated pre-QC

To facilitate the QC procedure, we ran a pre-QC analysis to automatically rate bundles according to pre-defined criteria before manual inspection. These criteria are detailed in Table 3. Afterwards, all bundles were looked at manually through an easier procedure that consists in confirming an already assigned rating rather than rating from scratch.

**Table 3:** Automatic rating criteria, in order of priority.

Rating	Criteria
Fail	*x* < 50
	*x* = 0 in either hemisphere (if symmetric bundle).
Warning	$x\notin [\mu -1.5\sigma ,\mu +3.5\sigma ]$
Pass	$x\in [\mu -1.5\sigma ,\mu +3.5\sigma ]$

*x* is the number of streamlines of the bundle of interest;

*μ* and *σ* are the average and the standard deviation, respectively, of the number of streamlines for the bundle of interest, across all subjects.

##### Manual quality control using *dmriqcpy*

A bundle was removed if it looked visually incomplete or if it deviated from the expected pathway. A poor bundle reconstruction might have an algorithmic cause, such as sub-optimal tracking parameters or improper registration in RBX. It might also have an anatomical cause, such as unknown or undisclosed neurological conditions. Furthermore, visually evaluating a bundle reconstruction is very subjective, and a rater’s evaluation can be affected by the time of day, duration of QC, or even the angle of visualization in the QC tool^63^. For all those reasons, and with the goal of establishing a gold standard for ML tractography methods, we chose to be somewhat severe in the rating of bundles, in order to minimize the number of false positives, even if that meant missing out some true positive data. After QC, we chose to ignore the following bundles from the atlas due to generalized reconstruction errors: AC, CC_Te, Fx, ICP, PC, SCP. From the initial 354 volumes, after all the processing steps and quality control, we were left with 284 volumes and associated bundles. The final atlas bundles used to build *TractoInferno* and evaluate future candidate tractograms are shown in Fig. 5.

## Data Records

Available data include T1W images, DTI metrics maps (FA/AD/MD/RD), DWI images with bvals/bvecs, fODF maps and fODF peaks, white matter/grey matter/csf masks, DWI SH maps (SH of order 6 fitted to the DWI signal, using the descoteaux07 SH basis^53^: https://dipy.org/documentation/1.3.0./theory/sh_basis/, and reference tractograms for the bundles described above, if a bundle reconstruction was possible for the subject.

The data is publicly available on the OpenNeuro platform at https://openneuro.org/datasets/ds003900/versions/1.1.1^64^.

## Technical Validation

This section describes how we used *TractoInferno* to train machine learning models for tractography, and how we assessed each model’s performance.

### Evaluation pipeline for candidate tractograms

When evaluating machine learning tractography algorithms, we focus on the volume covered by the recognized bundles (compared to the gold standard bundles). We make no assumptions about the ability to “explore” the brain outside the scope of the *TractoInferno* dataset. Consequently, we ignore anything that is not recognized as a candidate bundle, and do not try to categorize streamlines as valid or invalid connections.

Candidate bundles are extracted in the same way that we defined the gold standard bundles. First, we run RBX to extract candidate bundles from the candidate whole-brain tractogram. Candidate bundles are then converted to binary volume coverage masks. Finally, each candidate mask is compared against its corresponding gold standard bundle mask to compute evaluation metrics.

For each subject in the testset, and for each available bundle of the given subject, we extract the following evaluation metrics: Dice score, overlap and overreach. The scores are averaged over all subjects of the testset to provide final scores. Altogether, these metrics help better understand the performance of a candidate tractography algorithm.

The evaluation pipeline is available online (https://github.com/scil-vital/TractoInferno/) and should be used with the provided *TractoInferno* testset, along with the default RBX-flow models.

### RNN-based tractography

To gauge the performances of ML models trained on the *TractoInferno* dataset, we implemented an RNN model and the necessary framework to train it on a large-scale tractography database, which was used multiple times in published papers in the last few years, such as *Learn2Track*^18^, *DeepTract*^21^, and *Entrack*^20^. Using the base implementation, we can easily modify the last layer of the model and its loss function to mimic the mentioned RNN models, and a few more.

We chose the stacked Long Short-Term Memory (LSTM) network as the recurrent building block for conditional streamline prediction. The LSTM is a type of RNN designed specifically to handle long-term dependencies, with the ability to deal with exploding and vanishing gradient problems^65^.

#### Learn2track

*Learn2track*^18^ proposed an RNN model for tractography, where the output of the model at each timestep is a 3D vector, used as the next direction of the streamline. The predicted vector is then scaled to the chosen step size, in order to match the lengths of the target and prediction.

From the same idea, we implemented an LSTM for deterministic tractography. As in the original *learn2track* paper, we used the squared error loss function between the target and prediction. The loss for a single streamline *S* composed of *T* steps is the following squared error:

$${\mathscr{L}}(S)=-\mathop{\sum }\limits_{t=1}^{T}{\left\Vert {d}_{t}-{\widehat{d}}_{t}\right\Vert }^{2}$$

where *d*_*t*_ and ${\widehat{d}}_{t}$ are the target and predicted directions. This model is noted as *Det-SE*.

However, to accurately reflect that only the direction of the predicted vector is important (not the magnitude), we also performed an experiment where we minimized the negative cosine similarity between the target and predicted directions:

$${\mathscr{L}}(S)=-\mathop{\sum }\limits_{t=1}^{T}{\rm{\cos }}({\theta }_{t})=-\mathop{\sum }\limits_{t=1}^{T}\frac{{d}_{t}\cdot {\widehat{d}}_{t}}{\left\Vert {d}_{t}\right\Vert \left\Vert {\widehat{d}}_{t}\right\Vert }$$

where *θ*_*t*_ is the angle between *d*_*t*_ and ${\widehat{d}}_{t}$. This model is noted as *Det-Cosine*.

#### DeepTract

In the same spirit as *learn2track*, *DeepTract*^21^ is a recurrent model for probabilistic tractography. In this case, the model output is a distribution over classes, where each class corresponds to a direction on the unit sphere, i.e. a discrete conditional fODF.

As in the original paper, we implemented a cross-entropy loss function:

$${\mathscr{L}}(S)=-\mathop{\sum }\limits_{t=1}^{T}\mathop{\sum }\limits_{m=1}^{M}{y}_{tm}{\rm{\log }}\left({\widehat{y}}_{tm}\right)$$

where *M* is the number of classes, and *y*_*t*_ and ${\widehat{y}}_{t}$ are vectors of target and predicted class probabilities. Note that we did not use label smoothing as in the original paper, nor entropy-based tracking termination. This model is noted as *Prob-Sphere*.

#### Entrack

*Entrack*^20^ is a non-recurrent artificial neural network for probabilistic tractography. The model is instead a feed-forward neural network, but includes the previous streamline direction as prior information to guide the tracking process. The model outputs the parameters for a von Mises-Fisher distribution, i.e. a 3D unit-length vector for the mean, and a scalar concentration parameter. The distribution is analogous to a Gaussian distribution, but defined on the unit sphere instead of euclidean space.

We chose to apply the same general idea, using a recurrent network that predicts the parameters for a von Mises-Fisher distribution on a 3D sphere. We used the negative log-likelihood of the von Mises-Fisher distribution as the loss function:

$${\mathscr{L}}(S)=-\mathop{\sum }\limits_{t=1}^{T}{\rm{\log }}\left[C({\widehat{\kappa }}_{t}){\rm{\exp }}\left({\widehat{\kappa }}_{t}{\widehat{\mu }}_{t}^{{\rm{T}}}{d}_{t}\right)\right]$$

where the predicted parameters of the distribution are ${\widehat{\mu }}_{t}$ (a unit-length vector) and ${\widehat{\kappa }}_{t}$ (a scalar concentration parameter), and *d*_*t*_ is the target unit-length vector at step *t*. $C({\widehat{\kappa }}_{t})$ abbreviates the normalization constant associated with the distribution, defined as following in the 3-dimensional case:

$${C}_{3}(\kappa )=\frac{\kappa }{2\pi \left({e}^{\kappa }-{e}^{-\kappa }\right)}$$

Note that unlike the original method, we didn’t use an entropy maximization scheme to regularize the predicted distribution. This implementation is noted as *Prob-vMF*.

#### Gaussian distribution output

Following *Entrack* and the idea of predicting the parameters of a continuous probability distribution, we implemented another model, using a multivariate Gaussian distribution instead of a von Mises-Fisher distribution. This model outputs a 3D vector for the mean, and 3 scalars for the variance, (one in each dimension). We choose to use a diagonal covariance matrix, for stability, and do not output any values for covariance.

In the 3-dimensional case, the negative log-likelihood loss function is:

$${\mathscr{L}}(S)=-\mathop{\sum }\limits_{t=1}^{T}{\rm{\log }}\left[\frac{1}{\sqrt{{(2\pi )}^{3}| {\widehat{{\boldsymbol{\Sigma }}}}_{t}| }}{\rm{\exp }}\left(-\frac{1}{2}{\left({d}_{t}-{\widehat{\mu }}_{t}\right)}^{{\rm{T}}}{\widehat{\Sigma }}_{t}^{-1}\left({d}_{t}-{\widehat{\mu }}_{t}\right)\right)\right]$$

where $${{\boldsymbol{\Sigma }}}_{t}=\left[\begin{array}{lll}{\sigma }_{xt}^{2} & 0 & 0\\ 0 & {\sigma }_{yt}^{2} & 0\\ 0 & 0 & {\sigma }_{zt}^{2}\end{array}\right]$$ is the predicted diagonal covariance matrix at streamline step *t*. This model is noted as *Prob-Gaussian*.

#### Gaussian mixture distribution output

The previous Gaussian model outputs a single average direction which is appropriated in most cases. However, there may be cases of bundle fanning or forking where the single-mode assumption may be an issue. This is because the Gaussian probability density can only be spread over a large area.

As such, some regions may be better modelled with more than one location of higher density. To this end, we implemented a mixture density network^66^ using a mixture of 3 Gaussian distributions. For each Gaussian, the model outputs 1 mixture weight, a 3D vector for the mean, and 3 scalars for the variances (again, we fix the covariances to zero).

In the 3-dimensional case, using a mixture of 3 Gaussians, the negative log-likelihood loss function is:

$$\begin{array}{lll}{\mathscr{L}}(S) & = & -\mathop{\sum }\limits_{t=1}^{T}{\rm{\log }}\left[\mathop{\sum }\limits_{k=1}^{3}{\phi }_{kt}{\mathscr{N}}\left({d}_{t}| {\widehat{\mu }}_{kt},{\widehat{\Sigma }}_{kt}\right)\right]\\  & = & -\mathop{\sum }\limits_{t=1}^{T}{\rm{\log }}\left[\mathop{\sum }\limits_{k=1}^{3}{\phi }_{kt}\frac{1}{\sqrt{{(2\pi )}^{3}| {\widehat{{\boldsymbol{\Sigma }}}}_{kt}| }}{\rm{\exp }}\left(-\frac{1}{2}{\left({d}_{t}-{\widehat{\mu }}_{kt}\right)}^{{\rm{T}}}{\widehat{\Sigma }}_{kt}^{-1}\left({d}_{t}-{\widehat{\mu }}_{kt}\right)\right)\right]\end{array}$$

where *k* denotes the number of Gaussians in the mixture, and *ϕ*_*kt*_ is the mixture parameter for the Gaussian *k* at streamline step *t*. This model is noted as *Prob-Mixture*.

#### Implementation details

All models were composed of 5 hidden layers of 500 units, used dropout with a rate of 0.1, and a batch size of 50 000 streamline steps. We added skip connections from the input layer to all hidden layers, and from all hidden layers to the output layer, inspired by (Graves, 2013)^67^. We applied layer normalization^68^ between all hidden layers, in order to stabilize the hidden state dynamics in recurrent neural networks. We used the Adam optimizer with the default parameters.

For all experiments, we used the maximal spherical harmonics (SH) coefficients of order 6 fitted to the TractoFlow-processed DWI signal as the input signal, without any other pre-processing. In all cases, the models were trained using the exact same training and validation datasets, with streamlines resampled to a fixed step size of 1.0 mm. To help guide the model, we also included as input the diffusion signal in a neighbourhood of 6 directions (two for each axis, positive and negative) at a distance of 1.2 mm.

All models were trained for a maximum of 30 epochs (corresponding to around 2 weeks of training time on a 16 Gb NVidia V100SXM2), but early stopping was used to stop training when the loss has not improved after 5 epochs. Each epoch was capped to 10 000 updates, as the sheer size of the dataset would otherwise require multiple days of training for a single epoch.

### Baselines benchmark results

Machine learning models were trained using the *TractoInferno* database, with a training set of 198 volumes and a validation set of 58 volumes. We report in Table 4 the results of the *TractoInferno* evaluation pipeline on the testing set of 28 volumes. Results include each individual tractography algorithm used to build the reference bundles, along with predictions for every trained ML model.

**Table 4:** Tractography evaluation results on the *TractoInferno* dataset. The Prob-vMF model did not produce valid results, and is noted as {N/A}.

	Dice	Overlap	Overreach
*Reference methods*			
Deterministic	0.397	0.267	0.029
Probabilistic	0.553	0.433	0.068
PFT	0.680	0.688	0.266
SET	0.624	0.570	0.184
Ensemble (Det + Prob + PFT + SET)	1.000	1.000	0.000
*RNN-based methods*			
Det-SE (Learn2track)	0.580	0.495	0.172
Det-Cosine	0.606	0.535	0.204
Prob-Sphere (DeepTract)	0.601	0.534	0.202
Prob-vMF (Entrack)	N/A	N/A	N/A
Prob-Gaussian	0.624	0.585	0.264
Prob-Mixture	0.407	0.284	0.053

Of all the base algorithms used to build the reference tractograms, PFT performed the best in terms of Dice score and overlap. This is consistent with the fact that it is a state-of-the-art algorithm, and works best when trying to fill the space with streamlines. However, we show that no algorithm can single-handedly account for the gold standard, and using the union of all methods provides a more complete reconstruction.

In both traditional and RNN-based variants, models with the best Dice/ overlap results also had the worst overreach score. However, in the case of bundle reconstruction, it is less of a concern, because there is always a possibility of applying post-processing techniques to filter streamlines. Also, since our gold standard is not perfect, it might not cover the whole possible space as delineated by the RBX algorithm. Furthermore, because the scores are evaluated using binary bundle masks, a small number of streamlines can easily cross a high number of overreaching voxels. Ultimately, the goal is to find a model that can cover as much space as possible, so the overreach score is an interesting information to have, but is not the best indicator of performance in our case.

Of all the RNN-based methods, the Gaussian output model obtained the best Dice score and overlap, hinting that a probabilistic model works best. This is in line with traditional probabilistic algorithms being more suited to bundle reconstruction than deterministic approaches. Given the worse performance of other probabilistic models, it seems that adding complexity is not always beneficial. Training an RNN with a more complex distribution like the mixture of Gaussians might require a different architecture, or more model capacity, to achieve better results. Unfortunately, the RNN with a von Mises-Fisher output had a hard time training, and produced erratic streamlines that mostly did not survive the evaluation pipeline. It would seem that training the vMF distribution is too unstable when using a likelihood loss function, and performing an entropy maximization procedure like the original authors might be required to have a stable training procedure.

To evaluate the out-of-distribution generalization capabilities of ML models, we additionally ran leave-one-site-out cross-validation experiments. In this case, each model was trained on 5 sites out of 6, and tested on the unseen site. We repeated the process 6 times, each time using a new site as an independent testset, effectively running 30 additional experiments (5 models × 6 leave-one-out datasets). We report the mean and standard deviation of all evaluation metrics across the 6 experiments for each model in Table 5. Cross-validation results are overall very similar to whole-dataset training. Encouragingly, it would indicate a suitable robustness of ML tractography models to unseen scanners after training on as few as 5 different scanners.

**Table 5:** Tractography cross-validation results on the *TractoInferno* dataset. The Prob-vMF model did not produce valid results, and is noted as {N/A}.

	Dice	Overlap	Overreach
*Reference methods*			
Deterministic	0.409 ± 0.031	0.276 ± 0.027	0.027 ± 0.005
Probabilistic	0.571 ± 0.032	0.448 ± 0.031	0.066 ± 0.009
PFT	0.691 ± 0.078	0.692 ± 0.120	0.257 ± 0.062
SET	0.638 ± 0.044	0.590 ± 0.072	0.188 ± 0.047
Ensemble (Det + Prob + PFT + SET)	1.000 ± 0.000	1.000 ± 0.000	0.000 ± 0.000
*RNN-based methods*			
Det-SE (Learn2track)	0.610 ± 0.022	0.539 ± 0.032	0.206 ± 0.039
Det-Cosine	0.599 ± 0.043	0.528 ± 0.052	0.198 ± 0.030
Prob-Sphere (DeepTract)	0.550 ± 0.051	0.449 ± 0.052	0.128 ± 0.027
Prob-vMF (Entrack)	N/A	N/A	N/A
Prob-Gaussian	0.561 ± 0.049	0.475 ± 0.059	0.166 ± 0.041
Prob-Mixture	0.489 ± 0.056	0.381 ± 0.059	0.101 ± 0.033

Across all results (both reference algorithms and RNN-based methods, either whole-dataset training or cross-validation), the general trend holds that with a better Dice score and overlap, there is also more overreach. This indicates that there is still work to be done to limit the production of false positive streamlines.

**Fig. 6:**
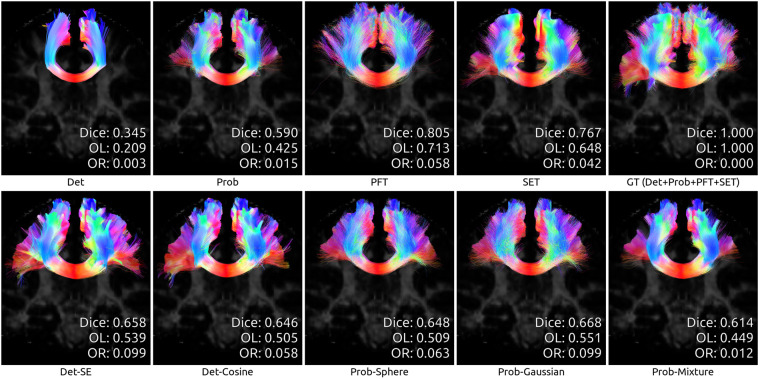
Reconstruction of the *Corpus Callosum* (medium difficulty) by all algorithms, for test subject sub-1006.

**Fig. 7:**
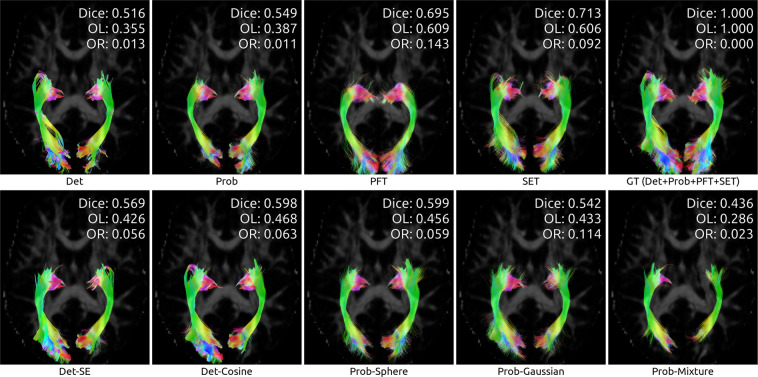
Reconstruction of the *Optic Radiation* (hard difficulty) by all algorithms, for test subject sub-1006.

**Fig. 8:**
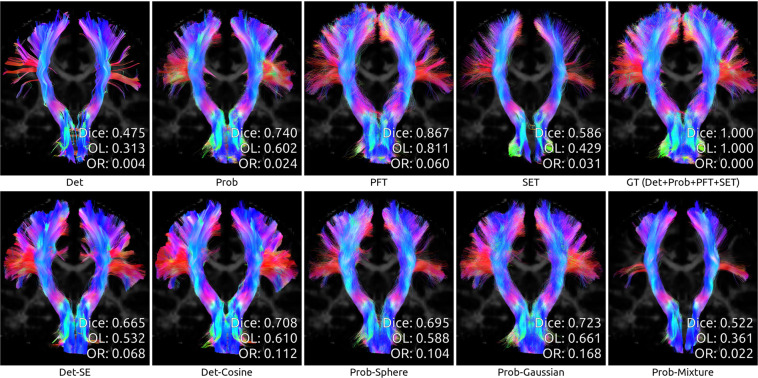
Reconstruction of the *Pyramidal Tract* (hard difficulty) by all algorithms, for test subject sub-1006.

To illustrate the differences between algorithms, we showcase the reconstructions of three bundles taken from a random test subject after whole-dataset training. We chose bundles of both *medium* and *hard* difficulty for tractography, as reported in (Maier-Hein *et al*.)^2^. Figure 6 shows a part of the *Corpus Callosum* (medium difficulty), while Figs. 7 and 8 show the *Optic Radiation* and the *Pyramidal Tract* (hard difficulty). Note that in all cases, as mentioned before, the Prob-vMF method did not produce any meaningful results, which explains why no results are shown.

Also of note, RNN-based models seem to get results on par with traditional algorithms, but not quite as good as the state-of-the-art Particle-Filtered Tractography. However, (Poulin *et al*.)^69^ produced results far beyond even PFT using an RNN approach trained on a single-database, using a single-bundle per model^69^. While we did not train any model with the single-bundle approach on *TractoInferno*, both results hint that there is a need for more data, more model capacity, or for specialization of algorithms, in order to outperform currently-used methods. We advocate that *TractoInferno* is one way to investigate this problem further.

In conclusion, ML tractography methods seem to reproduce (to a worse degree) what ensemble tractography finds. Possible reasons for this is that there is some noise in the gold standard streamlines used for training, and models may somewhat under-fit the data. Indeed, all standard tractography algorithms produce noisy approximations of possible white matter tracts. Furthermore, the bundle segmentation method used to produce gold standard bundles is far from perfect and can be variable from one execution to another, which affects both gold standard streamlines, and the evaluation procedure (however, it is still one the best methods available given the large-scale of TractoInferno). In addition, all ML models used in this paper were trained up to a hard time threshold of two weeks given limited computational resources, and some of those models had not yet attained a training loss minima, which points to under-fitted models. Given the still increasing computation capabilities of GPUs, future experiments would do well to train models up to completion, while also augmenting model capacity by increasing the size and number of hidden layers until one can reach overfitting conditions.

### Potential limitations

The proposed dataset and evaluation methods are not void of limitations. First, the bundle segmentation method (RecoBundlesX) is not perfect, and suffers from some degree of variability between executions, which affects both the gold standard bundles and the evaluation of candidate tractograms. Second, the *TractoInferno* dataset contains only healthy subjects; it is unclear how trained models might perform on unhealthy subjects, and should be used only with caution. Finally, we experimented with recurrent neural networks, while there are other model architectures that could provide useful for tractography, such as convolutional neural networks like TractSeg^19^ and Transformer models^70^.

## Usage Notes

The data available on OpenNeuro contains a /derivatives directory, which contains all processed data, organized into training, validation, and testing subsets. Files are organized first by subject (sub-*/), then by file type (e.g. anat/). All files follow the same naming convention: [SUBJECT_ID]__[FILENAME].[EXT].

Tractograms contain compressed streamlines to reduce space, which means that the step size is variable. If a fixed step size is required, it is possible to manually resample the streamlines, using the public repository SCILPY (https://github.com/scilus/scilpy) and the scil_resample_streamlines.py script, found here: https://github.com/scilus/scilpy/blob/master/scripts/scil_resample_streamlines.py .

**Table 6:** *TractoInferno* processing steps.

Processing step	Software/Tool	URL
Quality control (DWI, TractoFlow, RBX)	dmriqcpy	github.com/scilus/dmriqcpy
		github.com/scilus/dmriqc_flow
Data processing	TractoFlow	github.com/scilus/tractoflow/
SH signal fitting	SCILPY	github.com/ppoulin91/tractoinferno_compute_sh_flow
Tractography (Det, Prob, PFT)	SCILPY	github.com/ppoulin91/tractoinferno_tracking_flow
Tractography (SET)	SET	github.com/StongeEtienne/set-nf
		github.com/scilus/convert_set_flow
Bundle delineation	RecoBundlesX (RBX)	github.com/scilus/rbx_flow/

## Data Availability

All code used to process the database is publicly available online. References to software are provided in Table 6. The pipeline to evaluate new candidate tractograms along with the test set is available here: https://github.com/scil-vital/TractoInferno/, and should be used with the same reference atlas: https://zenodo.org/record/4630660#.YJvmwXVKhdU^62^.
